# One Pathway Is Not Enough: The Cabbage Stem Flea Beetle *Psylliodes chrysocephala* Uses Multiple Strategies to Overcome the Glucosinolate-Myrosinase Defense in Its Host Plants

**DOI:** 10.3389/fpls.2018.01754

**Published:** 2018-12-07

**Authors:** Franziska Beran, Theresa Sporer, Christian Paetz, Seung-Joon Ahn, Franziska Betzin, Grit Kunert, Anton Shekhov, Daniel G. Vassão, Stefan Bartram, Sybille Lorenz, Michael Reichelt

**Affiliations:** ^1^Research Group Sequestration and Detoxification in Insects, Max Planck Institute for Chemical Ecology, Jena, Germany; ^2^Research Group Biosynthesis/NMR, Max Planck Institute for Chemical Ecology, Jena, Germany; ^3^Department of Biochemistry, Max Planck Institute for Chemical Ecology, Jena, Germany; ^4^Department of Bioorganic Chemistry, Max Planck Institute for Chemical Ecology, Jena, Germany; ^5^Research Group Mass Spectrometry, Max Planck Institute for Chemical Ecology, Jena, Germany

**Keywords:** *Psylliodes chrysocephala*, glucosinolate, sequestration, detoxification, cabbage stem flea beetle, isothiocyanate, glutathione

## Abstract

The cabbage stem flea beetle (*Psylliodes chrysocephala)* is a key pest of oilseed rape in Europe, and is specialized to feed on Brassicaceae plants armed with the glucosinolate-myrosinase defense system. Upon tissue damage, the β-thioglucosidase enzyme myrosinase hydrolyzes glucosinolates (GLS) to form toxic isothiocyanates (ITCs) which deter non-adapted herbivores. Here, we show that *P. chrysocephala* selectively sequester GLS from their host plants and store these throughout their life cycle. In addition, *P. chrysocephala* metabolize GLS to desulfo-GLS, which implies the evolution of GLS sulfatase activity in this specialist. To assess whether *P. chrysocephala* can largely prevent GLS hydrolysis in ingested plant tissue by sequestration and desulfation, we analyzed the metabolic fate of 4-methylsulfinylbutyl (4MSOB) GLS in adults. Surprisingly, intact and desulfo-GLS together accounted for the metabolic fate of only 26% of the total ingested GLS in *P. chrysocephala*, indicating that most ingested GLS are nevertheless activated by the plant myrosinase. The presence of 4MSOB-ITC and the corresponding nitrile in feces extracts confirmed the activation of ingested GLS, but the detected amounts of unmetabolized ITCs were low. *P. chrysocephala* partially detoxifies ITCs by conjugation with glutathione via the conserved mercapturic acid pathway. In addition to known products of the mercapturic acid pathway, we identified two previously unknown cyclic metabolites derived from the cysteine-conjugate of 4MSOB-ITC. In summary, the cabbage stem flea beetle avoids ITC formation by specialized strategies, but also relies on and extends the conserved mercapturic acid pathway to prevent toxicity of formed ITCs.

## Introduction

Crucifer-feeding insects encounter a potent chemical defense that consists of two components, the glucosinolates (GLS) and the β-thioglucoside hydrolase myrosinase (Halkier and Gershenzon, 2006). GLS are water-soluble β-thioglucoside-*N*-hydroxysulfates with a variable amino acid-derived side chain and occur almost exclusively in Brassicales plants (Fahey et al., 2001; Agerbirk and Olsen, 2012). While GLS are non-toxic, their hydrolysis by the myrosinase liberates noxious isothiocyanates (ITCs) with strong reactivity toward thiol- and amine-groups in peptides and proteins (Brown and Hampton, 2011). GLS and myrosinases are stored separately in the plant and ITCs are released rapidly in damaged tissue (Matile, 1980; Andréasson and Jørgensen, 2003). Feeding experiments with generalist as well as specialist insect herbivores have demonstrated negative effects of ITCs on growth, development, and survival (Li et al., 2000; Agrawal and Kurashige, 2003; Barth and Jander, 2006). For example, dietary ITCs depleted cysteine and glutathione levels and activated the catabolism of insect proteins in *Spodoptera littoralis* larvae, thereby leading to reduced larval weight gain (Jeschke et al., 2015).

Some plant species produce so-called specifier proteins that affect the products of GLS hydrolysis by promoting the formation of nitriles, epithionitriles, and thiocyanates (Burow and Wittstock, 2009; Kuchernig et al., 2012). Compared to ITCs, these compounds are less toxic to herbivores (Lambrix et al., 2001; Burow et al., 2006), but nitriles also play a role in direct and indirect plant defense (Mumm et al., 2008). Interestingly, a nitrile-specifier protein (NSP) was also identified in the larval gut of the cabbage white butterfly, *Pieris rapae*, where it efficiently prevents ITC formation in the ingested plant tissue (Wittstock et al., 2004). Within Pieridae, NSP activity was detected exclusively in crucifer-feeding species, which, together with phylogenetic analyses, demonstrated that NSPs represent an evolutionary key innovation that enabled a host plant shift from Fabales to Brassicales plants (Wheat et al., 2007; Edger et al., 2015).

Besides specifier proteins, insects evolved several other mechanisms to overcome the GLS-myrosinase defense system (reviewed in Winde and Wittstock, 2011; Jeschke et al., 2015). Larvae of the diamondback moth, *Plutella xylostella*, secrete a sulfatase enzyme into the gut lumen to convert ingested GLS to stable desulfo-GLS which are not a substrate of myrosinase (Ratzka et al., 2002). This GLS detoxification mechanism evolved convergently in two other generalist herbivores, the desert locust *Schistocerca gregaria* and the whitefly *Bemisia tabaci*, but the corresponding sulfatase enzymes in these two species are so far unknown (Falk and Gershenzon, 2007; Malka et al., 2016). Crucifer-feeding sawfly larvae (e.g., *Athalia rosae*) rapidly sequester GLS into the hemolymph where they are metabolized, and then excreted (Müller and Wittstock, 2005; Opitz et al., 2011; Abdalsamee et al., 2014). The cabbage aphid, *Brevicoryne brassicae*, and the striped flea beetle, *Phyllotreta striolata*, also accumulate high amounts of host plant GLS in their bodies to assemble their own GLS-myrosinase system using an insect myrosinase (Kazana et al., 2007; Beran et al., 2014). However, not all crucifer-specialists can prevent ITC formation during feeding. The herbivorous leaf-mining drosophilid *Scaptomyza flava* avoids plant tissues with high GLS concentration, and evolved a specific glutathione *S*-transferase to efficiently detoxify ITCs by conjugation to the tripeptide glutathione (GSH) (Gloss et al., 2014; Humphrey et al., 2016). Glutathione conjugates of ITCs are metabolized via the conserved mercapturic acid pathway to cysteinylglycine (CysGly), cysteine (Cys), and *N*-acetylcysteine (NAC) conjugates in *S. flava*. The mercapturic acid pathway plays a key role in the detoxification of dietary ITCs in generalist lepidopteran herbivores and molluscs as well, but the pathway end products may differ between species (Schramm et al., 2012; Falk et al., 2014; Jeschke et al., 2017).

In this study, we focus on the cabbage stem flea beetle, *Psylliodes chrysocephala*, a notorious pest of oilseed rape in Northern Europe (Williams, 2010; Zimmer et al., 2014). Both adults and larvae cause considerable crop damage by feeding on leaves and mining in stems and petioles of winter oilseed rape seedlings, respectively (Godan, 1951; Williams, 2010; Zimmer et al., 2014). Previous studies revealed that *P. chrysocephala* accept only GLS-containing plants as food (Bartlet and Williams, 1991), and that GLS stimulate adult feeding (Bartlet et al., 1994). Moreover, volatile GLS hydrolysis products attract adults in the field (Bartlet et al., 1992). These findings demonstrate that *P. chrysocephala* is highly adapted to GLS-containing plants, but how this specialist overcomes the ITC-based defense in its host plants is unknown. To answer this question, we analyzed whether the cabbage stem flea beetle is able to prevent GLS breakdown to ITCs, or metabolizes ITCs to avoid their toxicity.

## Materials and Methods

### Plants and Insects

Seeds of *Brassica rapa* cv. “Yu-Tsai-Sum” were purchased from Known-You Seed (Taiwan). Plants were cultivated in a controlled environment chamber (21°C, 55% relative humidity, 14-h light/ 10-h dark period). *Arabidopsis thaliana* Col-0 wild-type, *A. thaliana myb28* × *myb29* double-knockout (DKO) mutant plants which are devoid of aliphatic glucosinolates (GLS) (Sønderby et al., 2007), and *A. thaliana tgg1* × *tgg2* DKO mutant plants lacking myrosinase activity (Barth and Jander, 2006), were cultivated under short-day conditions in a controlled environment chamber (21°C, 55% relative humidity, 10-h light/ 14-h dark period).

The laboratory culture of *P. chrysocephala* was established in 2012 from adults collected in Laasdorf, Thuringia, Germany. Adults were reared on potted three- to four-week old *B. rapa* plants in a controlled environment chamber (24°C, 75% relative humidity, 16-h light/8-h dark period). Plants were exchanged every week. Plants with eggs were kept in a separate cage for larval development. After 4 weeks, the remaining plant material was cut and the soil with pupae was kept in plastic containers (9 l volume, Lock&Lock). Newly emerged beetles were collected from the soil containers every two to three days and reared on potted plants as described above.

### Chemicals and Chemical Syntheses

4-Methylsulfinylbutyl (4MSOB) GLS was purchased from Phytoplan (Heidelberg, Germany) and 4-hydroxybenzyl (4OHBenzyl) GLS was isolated from seeds of *Sinapis alba* according to Thies (1988). GLS were converted to desulfo-glucosinolates using *Helix pomatia* sulfatase solution prepared according to Graser et al. (2001). Recombinant β-O-glucosidase from *Caldocellum saccharolyticum* was used to prepare 4MSOB cyanide from desulfo-4MSOB GLS as described in Wathelet et al. (2001). 4MSOB isothiocyanate (ITC) was purchased from Alexis Biochemicals (San Diego, USA). 4MSOB-amine, 4MSOB-ITC-glutathione (GSH), 4MSOB-ITC-cysteine (Cys), and 4MSOB-ITC-*N*-acetylcysteine were purchased from Santa Cruz Biotechnology (Dallas, USA). 4MSOB-ITC-cysteinylglycine (CysGly) was synthesized and purified as described by Kassahun et al. (1997) by using CysGly (Sigma-Aldrich, Munich, Germany) instead of GSH. 4MSOB-acetamide was synthesized from 4MSOB-amine as described by Song et al. (2005).

The conjugate of 4MSOB-ITC with U-^13^C and ^15^N-labeled L-cysteine (Sigma-Aldrich; purity 98%) was synthesized according to Kassahun et al. (1997) with the following modifications: 0.16 mmol of 4MSOB-ITC in 2.4 ml ethanol were mixed with 0.16 mmol U-^13^C^15^N-L-cysteine dissolved in 3.4 ml 50% (v/v) ethanol (pH 7.5). The mixture was stirred overnight under nitrogen atmosphere at room temperature. The 4MSOB-ITC-^13^${\text{C}}_{3}^{15}$N-Cys conjugate was purified by preparative HPLC using Agilent 1100 series equipment (Agilent Technologies, Böblingen, Germany) using a Supelcosil LC-18-DB Semi-Prep column (250 × 10 mm, 5 μm particle size, Supelco, Bellefonte, USA). Separation was accomplished using a mobile phase consisting of water as solvent A and acetonitrile as solvent B, with the flow rate set at 4 ml/min. The gradient was as follows: 10% (v/v) B (0.5 min), 10–30% (v/v) B (6.5 min), 30–100% (v/v) B (0.1 min), 100% B (v/v) (2.4 min), 100–10% (v/v) B (0.1 min), 10% (v/v) B (2.4 min), compound retention time (RT) 5.3 min.

The cyclic 4MSOB-ITC conjugate 2-(4-(methylsulfinyl)butylamino)-4,5-dihydrothiazole-carboxylic acid (4MSOB-ITC-Cyclic-Cys conjugate A) was synthesized by stirring a mixture of 0.1 mmol 4MSOB-ITC dissolved in 0.35 ml ethanol and 0.1 mmol of L-cysteine dissolved in 4.65 ml water (pH 5.8) at 80°C for 2 h. The cyclic product was isolated by preparative HPLC on a Supelcosil LC-18-DB Semi-Prep column (250 × 10 mm, 5 μm particle size, Supelco, Bellefonte, USA) using a mobile phase consisting of 0.05% (v/v) formic acid in water as solvent A and acetonitrile as solvent B, with the flow rate set at 4 ml/min. The gradient was as follows: 5–15.5% (v/v) B (7 min), 15.5–100% (v/v) B (0.1 min), 100% (v/v) B (1.9 min), 100–5% (v/v) B (0.1 min), 5% (v/v) B (3.9 min), compound RT 6.1 min.

The cyclic 4MSOB-ITC conjugate 4-hydroxy-3-(4-(methylsulfinyl)butyl)-2-thioxothiazolidine-4-carboxylic acid (4MSOB-ITC-Cyclic-Cys conjugate C) was synthesized by stirring a mixture of 14.1 μmol 4MSOB-ITC in 0.5 ml ethanol and 16.8 μmol mercaptopyruvate (Sigma-Aldrich; purity ≥97%) in 0.5 ml 50% (v/v) ethanol under nitrogen atmosphere at room temperature overnight. The product was purified by solid phase extraction (SPE) using a Chromabond® C_18_ec column (500 mg, Macherey-Nagel, Düren, Germany) followed by preparative HPLC using an EC 250/4.6 Nucleodur Sphinx RP column (250 × 4.6 mm, 5 μm particle size; Macherey-Nagel, Düren, Germany). The mobile phase consisted of 0.05% (v/v) formic acid in water as solvent A and acetonitrile as solvent B, with the flow rate set at 1 ml/min. The gradient was as follows: 5–41% (v/v) B (12 min), 41–100% (v/v) B (0.1 min), 100% (v/v) B (2.9 min), 100–5% (v/v) B (0.1 min), 5% (v/v) B (3.9 min), compound RT 10.5 min.

### Sampling of *Brassica rapa* and Different Life Stages of *P. chrysocephala*

Leaves of intact four-week old *B. rapa* plants with six to seven true leaves, and of four-week old plants that had been damaged by *P. chrysocephala* adults for one week were harvested to analyze their GLS profiles (*N* = 5–8). From each plant, the second, third, and fourth fully expanded leaf was harvested separately, weighed, frozen in liquid N_2_, and freeze-dried. To analyze GLS in *P. chrysocephala*, eggs, larvae (stages L2 and L3), prepupae, pupae, and adults (newly emerged, one-, three-, seven-, and 14-day old fed on *B. rapa*) were collected from the laboratory rearing, weighed, frozen in liquid N_2_ and stored at −20°C until extraction (*N* = 7–9). Eggs were washed three times with water before they were frozen.

### GLS Extraction and Analysis

The extraction and analysis of GLS from homogenized *B. rapa* leaves and *P. chrysocephala* samples was performed as described in Beran et al. (2014). 4OHBenzyl GLS was used as internal standard and desulfo-GLS were eluted from DEAE-Sephadex columns with 0.5 ml ultrapure water. Samples were stored at −20°C until analysis by HPLC-UV at 229 nm.

### Data Analysis

The GLS concentrations in intact and damaged *B. rapa* plants were determined as the average GLS concentration of the three leaves per plant. The concentrations of individual GLS in undamaged and feeding-damaged plants were compared using Student's *t*-test or Mann-Whitney *U*-test if data were not normally distributed. The GLS profiles of intact and damaged *B. rapa* leaves and *P. chrysocephala* at different life stages were analyzed by principal component analysis (PCA) using the software MetaboAnalyst 3.0 (Xia and Wishart, 2011, 2016). Total GLS concentration was set to 100%, and the percentage of each GLS was calculated. Prior to analysis, data were cube-root transformed and mean centered. The result was visualized in score and loadings plots. Ellipsoids in the score plot correspond to the 95% confidence intervals of each group. Total GLS amount per individual, total GLS concentration, and percentages of individual GLS were compared using either analyses of variance (ANOVA) followed by the *post-hoc* Tukey HSD test, gamma generalized linear model, or the method of generalized least squares (nlme library, Pinheiro et al., 2017) depending on the variance homogeneity and the normality of residuals. If necessary, data were arcus-sinus-square-root transformed in order to achieve normality of residuals. For data analyzed with the generalized least squares method, the varIdent variance structure (which allows each group to have a different variance) was applied. *P*-values were obtained by removing the explanatory variable and comparison of models with likelihood ratio test (Zuur et al., 2009). To analyze which groups differed from each other, factor level reduction was applied (Crawley, 2013). Analyses were done in Sigma Plot 11.0 or in R 3.4.1 (R Core Team, 2017). Information about transformation, used method, and result of the statistical analysis for each GLS is summarized in Supplementary Table S1.

### Quantitative Feeding Experiment With 4MSOB GLS

To analyze the metabolic fate of ingested GLS in *P. chrysocephala* adults, a quantitative feeding experiment was performed using 4MSOB GLS that is not present in the host plant *B. rapa*. A total of 250 nmol 4MSOB GLS were incorporated into detached leaves of the *A. thaliana myb28* × *myb29* DKO mutant as described in Schramm et al. (2012). Leaves were placed individually in 5 ml tubes (Eppendorf, Germany) with four newly emerged adults (*N* = 6). Beetles fed with an *A. thaliana myb28* × *myb29* DKO mutant leaf without additional GLS served as background control (*N* = 3), and leaves without beetles were used to determine the GLS recovery rate under feeding assay conditions (*N* = 4). After feeding for one day, the beetles were transferred to a new tube containing a non-spiked *A. thaliana myb28* × *myb29* DKO mutant leaf for an additional day to ensure that all plant material containing 4MSOB GLS had been digested and excreted. Remaining leaves were frozen in liquid N_2_ and freeze-dried. Feces were collected in 0.1% (v/v) formic acid in water, and stored at −20°C until extraction. Beetle and leaf samples were homogenized, extracted using 1 ml 50% (v/v) methanol in 0.1% (v/v) formic acid, and transferred to glass vials after centrifugation. Feces samples were homogenized and centrifuged, and the supernatant was transferred to a glass vial. Extracts of feces and the second non-spiked leaf were evaporated to dryness using a gentle nitrogen stream, re-dissolved in 100 μl of 50% (v/v) methanol in 0.1% (v/v) formic acid, and stored at −20°C until LC-MS/MS analysis.

### Sequestration Experiment With *A. thaliana tgg1* × *tgg2* and Col-0 Wild-ype

A feeding experiment was performed to compare the accumulation of intact 4MSOB GLS in beetles which fed on the myrosinase-deficient *tgg1* × *tgg2* mutant and on Col-0 wild-type leaves, respectively. Two newly emerged adults were placed in a Petri dish with a moistened filter paper and a cut *A. thaliana* leaf from a five-week old plant (*N* = 24). On the next day, the remaining leaf tissue was weighed, frozen in liquid N_2_ and freeze-dried. Adults were supplied with a *myb28* × *myb29* leaf until they were weighed and frozen in liquid N_2_ on the next day. GLS present in leaves and adults were extracted and analyzed as described above. The 4MSOB GLS concentration in adults was determined as percentage relative to the 4MSOB GLS concentration in the corresponding leaf (set to 100%). The 4MSOB GLS concentration in the fed leaves of the two *A. thaliana* genotypes was compared by Student's *t*-test, and the relative accumulation of 4MSOB GLS in beetles was compared by Mann-Whitney *U*-test in Sigma Plot 11.0. To test whether beetles feed equally on the two different *A. thaliana* genotypes, the remaining leaf area after feeding of two newly emerged adults on a leaf disc (16 mm diameter) for five hours was analyzed using the software Fiji (Schindelin et al., 2012) (*N* = 27). The remaining leaf area was compared by Student's *t*-test in Sigma Plot 11.0.

### Identification of 4MSOB GLS-Derived Metabolites in Feces of *P. chrysocephala*

To identify other 4MSOB GLS-derived metabolites, feces were analyzed after adults fed on cut leaves of *A. thaliana* Col-0 wild-type plants, leaves of the *tgg1* × *tgg2* and *myb28* × *myb29* DKO mutants, and *myb28* × *myb29* leaves spiked with 4MSOB GLS as described above, respectively (*N* = 3). Feces of six newly emerged adults that fed on one leaf for two days were collected and extracted in 200 μl 0.1% (v/v) formic acid (pH 3). Samples were stored at −20°C until HPLC-MS (ion trap) analysis.

### *In vivo* Metabolism of 4MSOB-Amine and the 4MSOB-ITC-^13^${\text{C}}_{3}^{15}$N-Cys-conjugate in Adults

To elucidate the metabolic origin of previously unknown 4MSOB-ITC-derived metabolites which were identified in feces of *P. chrysocephala*, adults were forced to ingest 0.2 μl of 4MSOB-amine (74 mM), and 4MSOB-ITC-^13^${\text{C}}_{3}^{15}$N-Cys conjugate (33.5 mM), respectively (*N* = 3). Feces of five to ten beetles per replicate were collected after beetles fed on a cut *myb28* × *myb29* leaf for one day. Samples were extracted and stored as described in the previous section.

### Identification and Qualitative Analyses of 4MSOB-ITC-Derived Metabolites in Aqueous Feces Samples by HPLC-MS

Comparative HPLC-MS (ion trap) analyses of aqueous feces extracts were carried out on an Agilent 1100 series HPLC (Agilent Technologies, Waldbronn, Germany) coupled to an Esquire 6000 ESI-Ion Trap mass spectrometer (Bruker Daltonics, Bremen, Germany) operated in alternating ionization mode in the range *m/z* 55–1000. Skimmer voltage −40 eV, capillary exit voltage −102.3 eV, capillary voltage 4,000 V, nebulizer pressure 35 psi, drying gas, 11 l/min; gas temperature 330°C. MS^2^ and MS^3^ spectra of candidate metabolites were obtained using the AutoMS mode of the software (Bruker Daltonics). Separation was accomplished using a Nucleodur Sphinx RP column (250 × 4.6 mm, 5 μm particle size, Macherey-Nagel) with a mobile phase consisting of 0.2% (v/v) formic acid in ultrapure water as solvent A and acetonitrile as solvent B with a flow rate of 1.0 ml/min at 25°C. The gradient was as follows: 0–100% (v/v) B (25 min), 100% B (3 min), 100–0% (v/v) B (0.1 min), 0% B (4.9 min). Eluent flow rate was diverted in a ratio of 4:1 before entering the ESI unit. Chromatograms were analyzed with the DataAnalysis and MetaboliteTools software packages from Bruker Daltonics. Candidate metabolites were further analyzed using MS/MS and high resolution mass spectrometry (see section UHPLC-ESI-MS/MS).

### UHPLC-ESI-MS/MS

To determine the exact mass of candidate metabolites, ultra-high-performance liquid chromatography–electrospray ionization–tandem mass spectrometry (UHPLC–ESI–MS/MS) was performed with an Ultimate 3000 series RSLC (Dionex, Sunnyvale, CA, USA) and a LTQ-Orbitrap XL mass spectrometer (Thermo Fisher Scientific, Bremen, Germany). UHPLC was used applying an Acclaim C18 column (150 × 2.1 mm, 2.2 μm particle size, Dionex). Separation was accomplished using a mobile phase consisting of 0.1% (v/v) formic acid in ultrapure water as solvent A and 0.1% (v/v) formic acid in acetonitrile as solvent B with a flow rate of 300 μl/min at 25°C. The gradient was as follows: 0–100% (v/v) B (15 min), 100% B (5 min), 100–0% (v/v) B (0.1 min), 0% B (5 min). ESI source parameters were set to 4,000 V for spray voltage, 35 V for transfer capillary voltage at a capillary temperature of 275°C. Samples were measured in positive and negative ionization mode at a mass range of *m/z* 50–1000 using 30,000 m/Δm resolving power in the Orbitrap mass analyzer. Data were evaluated and interpreted using XCALIBUR software (Thermo Fisher Scientific).

### Isolation of 4MSOB-ITC-Derived Metabolites Using Preparative HPLC

Three previously unknown 4MSOB-ITC-derived metabolites (*m/z* = 178 [M+H]^+^, RT 6.1 min; *m/z* = 295 [M-H]^−^, RT 7.2 min; *m/z* = 296 [M-H]^−^, RT 10.8 min) were isolated from feces extracts to elucidate their structure by nuclear magnetic resonance (NMR) spectroscopy. To obtain enough material for isolation, feces of 250 *P. chrysocephala* adults, which had fed on *A. thaliana* Col-0 wild-type plants for two consecutive days were collected and extracted in 0.1% (v/v) formic acid in water. The crude extract was purified and concentrated by SPE using a Chromabond® C_18_ec column (500 mg, Macherey-Nagel). The fraction obtained from elution with 20% (v/v) methanol was further fractionated by preparative HPLC using the method described for 4MSOB-ITC-Cyclic-Cys conjugate C. HPLC fractions were purified and concentrated by SPE and lyophilized. During the purification process we observed a spontaneous conversion of target metabolite *m/z* = 295 to metabolite *m/z* = 296 (4MSOB-ITC-Cyclic-Cys conjugate C). To obtain sufficient material of metabolite *m/z* = 295 for NMR, another feces extract was freeze-dried (ca. 40 mg feces dry weight) and extracted four times with 0.5 ml ultrapure water. Afterwards, a liquid-liquid extraction of the aqueous extract with dichloromethane was performed to remove 4MSOB-cyanide which co-eluted with the target metabolite. The final extract was fractionated by preparative HPLC and freeze-dried as described above. This procedure could not fully prevent the conversion of *m/z* = 295 to *m/z* = 296, but yielded a higher proportion of the target metabolite compared to the first attempt.

### Structure Elucidation Using NMR Spectroscopy

NMR spectra were recorded on a Bruker Advance III HD 700 spectrometer, equipped with a cryoplatform and a 1.7 mm TCI microcryoprobe (Bruker Biospin GmbH, Rheinstetten, Germany). NMR tubes of 1.7 mm outer diameter were used for all measurements. All NMR spectra were recorded using MeOH-*d*_3_ as a solvent. Chemical shifts were referenced to the residual solvent peaks at δ_H_ 3.31 and δ_C_ 49.15, respectively. Data acquisition and processing were accomplished using TopSpin 3.2. (Bruker Biospin). Standard pulse programs as implemented in TopSpin were used for data acquisition.

### Quantification of 4MSOB GLS and Derived Metabolites by LC-MS/MS

GLS and derived metabolites present in samples from the quantitative feeding experiment were quantified by liquid chromatography-tandem mass spectrometry (LC-MS/MS) using an Agilent 1200 HPLC system connected to an API3200 tandem mass spectrometer (AB Sciex, Darmstadt, Germany). Intact and desulfo-GLS were separated on a Nucleodur Sphinx RP column (250 × 4.6 mm, 5 μm particle size; Macherey-Nagel) using a mobile phase consisting of 0.2% (v/v) formic acid in ultrapure water as solvent A and acetonitrile as solvent B, at a flow rate of 1 ml/min. The gradient was as follows: 1.5% (v/v) B (1 min), 1.5–5% (v/v) B (5 min), 5–7% (v/v) B (2 min), 7–13.3% (v/v) B (4.5 min), 13.3–100% (v/v) B (0.1 min), 100% B (0.9 min), 100–1.5% (v/v) B (0.1 min), 1.5% (v/v) B (3.9 min). The presence of GLS was detected with the ionization source set to negative mode. Ionspray voltage was maintained at −4,500 eV. Gas temperature was set to 700°C, nebulizing gas to 70 psi, drying gas to 60 psi, curtain gas to 20 psi, and collision gas to 10 psi. Desulfo-GLS were detected in positive ionization mode. Ionspray voltage was maintained at 5,000 eV. Gas temperature was set to 700°C, nebulizing gas and drying gas to 60 psi, curtain gas to 35 psi, and collision gas to 5 psi. Hydrolysis products of 4MSOB GLS were separated on a Zorbax Eclipse XDB-C18 column (50 × 4.6 mm, 1.8 μm particle size; Agilent) using a mobile phase consisting of 0.05% (v/v) formic acid in ultrapure water as solvent A and acetonitrile as solvent B, at a flow rate of 1.1 ml/min. Separation of 4MSOB GLS hydrolysis products was achieved by using the following gradient: 3–15% (v/v) B (0.5 min), 15–85% (v/v) B (2 min), 85–100% (v/v) B (0.1 min), 100% B (0.9 min) 100–3% (v/v) B (0.1 min), 3% (v/v) B (2.4 min). Multiple-reaction monitoring (MRM) was used to monitor analyte parent ion-to-product ion formation (Supplementary Table S2). Compounds were quantified via external calibration curves (for sources of standards see section Chemicals and Chemical Syntheses). Data analysis was performed using Analyst Software 1.6 Build 3773 (AB Sciex).

### Myrosinase Activity Assay

Crude protein extracts of L3 larvae and adults (14 days old and starved for one day) were analyzed for myrosinase activity. For each sample, three to four individuals were pooled, the fresh weight was determined, and the tissue was immediately frozen in liquid N_2_. Three samples were prepared for each life stage and were stored at −80°C until protein extraction. The tissue (ca. 15 mg) was homogenized in 600 μl chilled 20 mM 2-(N-morpholino)ethanesulfonic acid (MES) buffer (pH 6.5) supplemented with protease inhibitors (cOmplete EDTA-free; Roche) using a 2-ml potter tissue grinder. The crude extract was ultracentrifuged at 4°C for 45 min at 48,000 × *g*. To remove intact GLS and other compounds that might interfere with the enzyme activity assay, the supernatant was first applied to a 20 mg DEAE-Sephadex A-25 column equilibrated with extraction buffer. The flow-through was subsequently transferred to a Zeba™ Spin Desalting Column (7KDa MWCO 2 ml; Thermo Scientific) equilibrated with extraction buffer. Afterwards, the protein concentration was determined using the Bradford protein assay (Bio-Rad) according to the manufacturer's manual. Myrosinase activity assays were performed with two different GLS as substrates: allyl GLS (Roth) and 2-hydroxy-3-butenyl GLS (Phytoplan, Heidelberg, Germany). Enzyme assays were performed in dark 96-well plates using the Amplite Fluorimetric Glucose Quantitation Kit (AAT Bioquest, Sunnyvale, USA). Each assay consisted of 25 μl protein sample (1.25 μg protein), 25 μl substrate at 2 mM (dissolved in MES buffer, pH 6.5) and 50 μl assay reagent. Assays without substrate and without protein, respectively, served as background control. Two technical replicates were measured for each substrate and the corresponding controls. Fluorescence intensity (Ex/Em = 540 nm/590 nm) was monitored every min for 30 min using a Tecan Infinite 200 Reader (Crailsheim, Germany). Glucose amounts in each assay were calculated from a glucose standard curve (dissolved in MES buffer, pH 6.5) for each time point. The amount of released glucose in the activity assay was determined by subtracting the amount of glucose detected in both background controls.

## Results

### Glucosinolates Are Present in All Life Stages of *P. chrysocephala*

**Figure 1:**
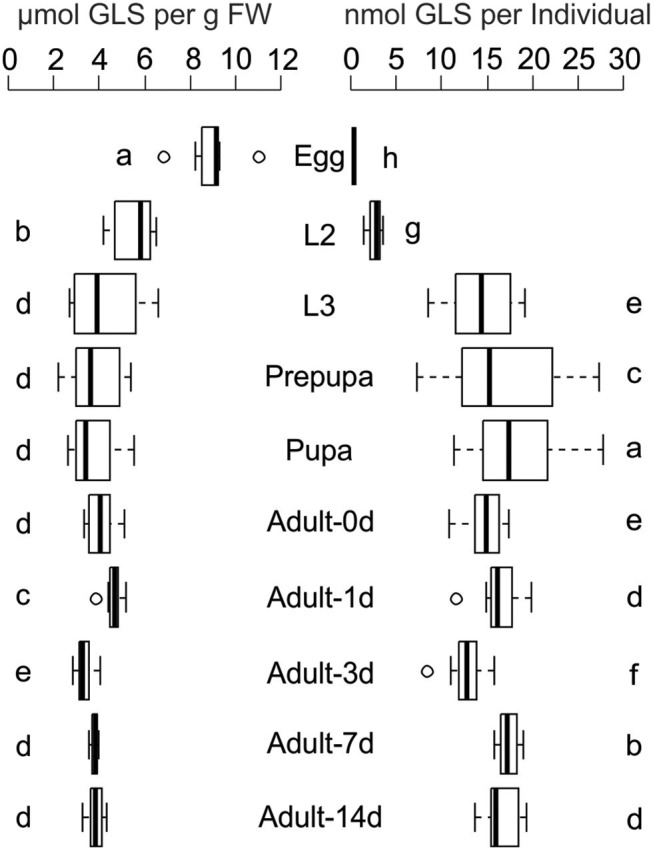
Glucosinolate (GLS) concentration and GLS amount per individual in all life stages of *P. chrysocephala* reared on *B. rapa* plants. Extracted GLS were analyzed by HPLC-UV, and GLS concentration is expressed per g fresh weight (FW). Different letters indicate significant differences between the life stages (*N* = 7–9). L2, second larval instar; L3, third larval instar. Details of statistical analyses are shown in Supplementary Table S1.

Chemical analyses of *P. chrysocephala* eggs, larvae, pupae, and adults revealed that GLS are present in all life stages at up to three-fold higher concentrations than detected in their rearing plant *B. rapa* (Figure 1; Table 1). GLS concentrations were similar in L3 larvae, pupae and adults (3.9 ± 0.9 μmol per g FW), but were significantly higher in eggs and L2 larvae (8.9 ± 1.3 and 5.5 ± 1.0 μmol GLS per g FW, respectively; *N* = 7–9; Figure 1; Supplementary Table S1).

**Table 1:** Glucosinolate (GLS) profile of intact and feeding-damaged four-week old *B. rapa*.

**GLS**	**Mean GLS concentration in** ***B. rapa*** **leaves [μmol × g**^****−1****^ **plant fresh weight ± SD]**			
	**Intact (*N* = 8)**	**Feeding-damaged (*N* = 5)**	**Statistics**	***P*-value**
3But	0.42 ± 0.25	0.29 ± 0.14	*t* = 1.080	*P* = 0.302
4Pent	0.62 ± 0.29	0.60 ± 0.28	*t* = 0.089	*P* = 0.931
2OH3But	0.30 ± 0.10	0.89 ± 0.72	*z* = −1.683	*P* = 0.093
2OH4Pent	0.05 ± 0.02	0.19 ± 0.18	*z* = −2.415	*P* = 0.015
5MSOP	0.04 ± 0.01	0.07 ± 0.05	*z* = −0.659	*P* = 0.510
5MTP	0.01 ± 0.01	0.01 ± 0.01	*z* = −0.220	*P* = 0.826
Benzyl	0.00 ± 0.00	0.07 ± 0.08	*z* = −3.003	*P* = 0.003
2PE	0.06 ± 0.02	0.06 ± 0.03	*t* = −0.367	*P* = 0.720
I3M	0.03 ± 0.01	0.15 ± 0.11	*z* = −2.855	*P* = 0.004
4OHI3M	0.02 ± 0.01	0.03 ± 0.02	*z* = 0.366	*P* = 0.714
4MOI3M	0.02 ± 0.01	0.02 ± 0.01	*t* = −0.517	*P* = 0.615
1MOI3M	0.01 ± 0.01	0.33 ± 0.45	*z* = −2.708	*P* = 0.007
Total	1.57 ± 0.53	2.70 ± 1.96	*z* = −1.244	*P* = 0.213

*GLS: 3But, 3-butenyl; 4Pent, 4-pentenyl; 2OH3But, 2-hydroxy-3-butenyl; 2OH4Pent, 2-hydroxy-4-pentenyl; 5MSOP, 5-methylsulfinylpentyl; 5MTP, 5-methylthiopentyl; 2PE, 2-phenylethyl; I3M, indol-3-ylmethyl; 4OHI3M, 4-hydroxyindol-3-ylmethyl; 4MOI3M, 4-methoxyindol-3-ylmethyl; 1MOI3M, 1-methoxyindol-3-ylmethyl*.

During development, the GLS amount per individual increased from 0.36 ± 0.04 nmol per egg to 18.4 ± 5.8 nmol in the pupa. The total GLS amount slightly decreased to 14.7 ± 2.3 nmol in newly emerged adults and remained at comparable levels in one-, three-, seven-, and 14-days old beetles which fed on *B. rapa* (*N* = 7–9; Figure 1; Supplementary Table S1).

**Figure 2:**
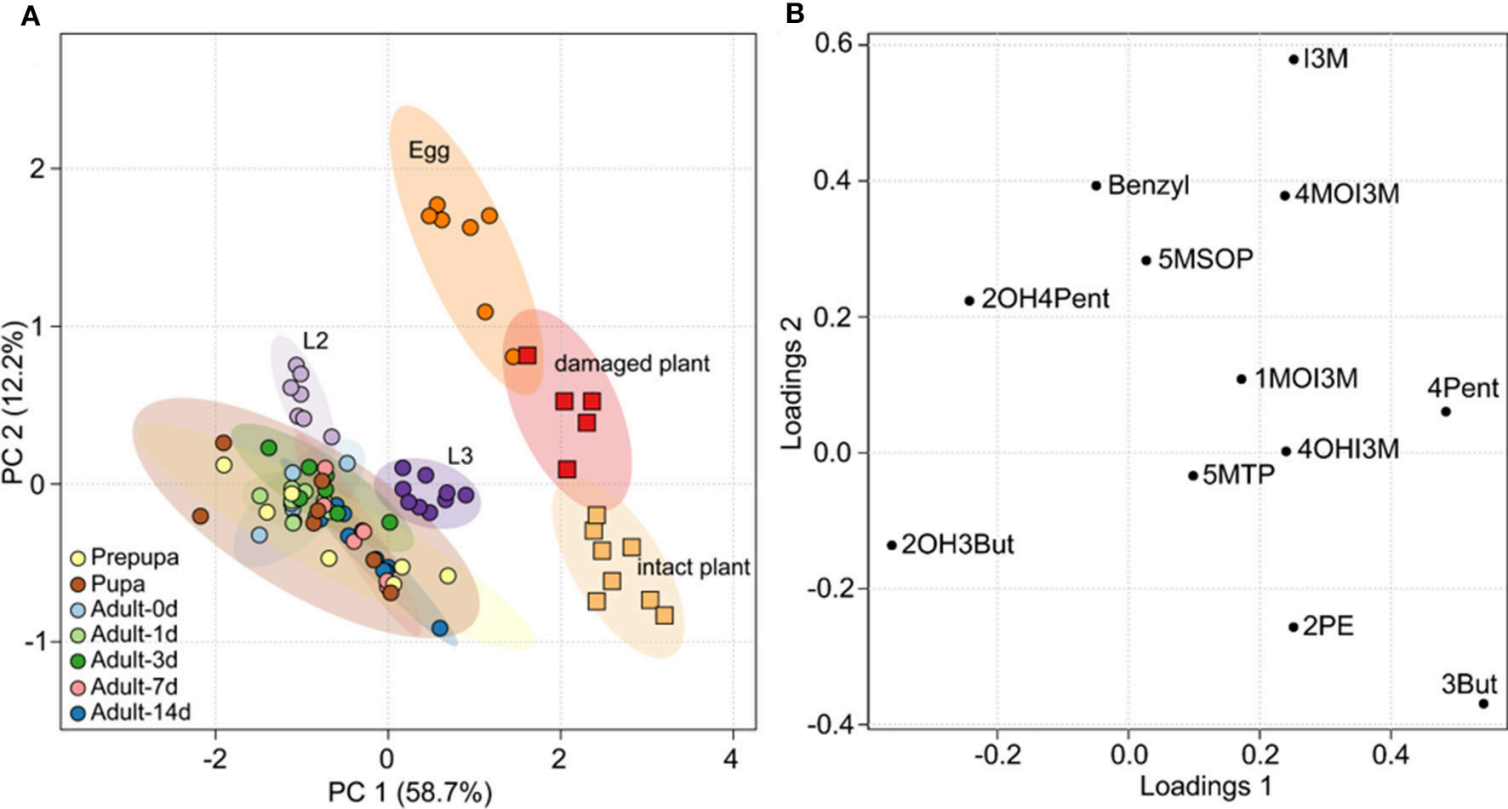
Principal component analysis (PCA) of the GLS composition in *B. rapa* and *P. chrysocephala*. GLS were extracted from intact and feeding damaged *B. rapa* leaves, and from all different life stages of *P. chrysocephala* (*N* = 5–9). GLS were analyzed by HPLC-UV and the percentage of each individual GLS was calculated from the total GLS concentration in each sample (Table 2). **(A)** PCA score plot; samples from *P. chrysocephala* are shown as circles, plant samples are shown as squares. Each circle or square represents the relative composition of all GLS in one replicate. The ellipsoids correspond to the 95% confidence intervals of each group. The GLS composition in *P. chrysocephala* larvae, prepupae, pupae and adults is very similar, but is separated from eggs and plants along principal component 1 (PC 1) which explains 58.7% of the total variability in the dataset. **(B)** PCA loadings plot showing the contribution of the individual GLS to the separation of the groups. 2-Hydroxy-3-butenyl-GLS (2OH3But), 3-butenyl GLS (3But), and 4-pentenyl GLS (4Pent) have the greatest influence on the separation of the groups along PC1. For abbreviations of GLS see legend of Table 1.

All GLS detected in insect samples were also present in the host plant, but the relative composition of GLS in *B. rapa* and *P. chrysocephala* differed significantly (Figure 2A; Table 2, Supplementary Table S1). Eggs had a distinct GLS composition resembling that of the damaged plant, and were separated from all other developmental stages, which clustered together in a principal component analysis (Figure 2A). Later life stages were separated from plants and eggs because they contained a higher proportion of 2-hydroxy-3-butenyl GLS (up to 75%), and significantly less 3-butenyl-, and 4-pentenyl GLS (Figure 2B).

**Table 2:** Composition of GLS profiles of intact and feeding damaged *B. rapa* leaves and different life stages of *P. chrysocephala*.

**GLS**	**GLS proportions (Mean percentage ± SD)^1^**											
	**Intact leaf (*N* = 8)**	**Damaged leaf (*N* = 5)**	**Egg (*N* = 7)**	**L2 (*N* = 7)**	**L3 (*N* = 9)**	**Prepupa (*N* = 7)**	**Pupa (*N* = 7)**	**Adult 0d (*N* = 7)**	**Adult 1d (*N* = 7)**	**Adult 3d (*N* = 7)**	**Adult 7d (*N* = 7)**	**Adult 14d (*N* = 9)**
3But	25.3 ± 8.6 a^2^	11.9 ± 3.2 b	2.8 ± 1.3 d	*0.1 ± 0.2*^3^	5.1 ± 2.2 c	2.6 ± 3.4 e	2.3 ± 2.9 e	1.1 ± 0.5 e	1.3 ± 0.8 e	0.9 ± 1.4 e	1.5 ± 1.1 e	2.4 ± 2.0 e
4Pent	37.3 ± 9.9 a	26.5 ± 10.0 b	23.3 ± 5.6 b	6.5 ± 1.5 d	12.7 ± 3.4 c	9.2 ± 9.4 d	5.9 ± 4.7 e	4.6 ± 3.8 e	3.7 ± 1.3 e	8.4 ± 2.8 d	11.5 ± 2.4 c	11.4 ± 4.0 c
2OH3But	21.4 ± 8.5 d	31.0 ± 6.1 c	40.1 ± 5.5 c	65.2 ± 2.4 ab	56.5 ± 4.5 b	69.9 ± 8.3 a	70.8 ± 5.4 a	74.8 ± 7.8 a	73.4 ± 4.0 a	74.7 ± 5.4 a	73.2 ± 2.4 a	72.9 ± 4.2 a
2OH4Pent	3.4 ± 1.6 d	6.6 ± 1.6 c	10.2 ± 3.7 c	19.4 ± 3.4 a	16.3 ± 3.8 b	14.8 ± 5.0 b	18.3 ± 8.3 a	15.4 ± 3.8 b	16.5 ± 3.9 b	9.4 ± 3.7 c	8.2 ± 3.6 c	7.6 ± 2.7 c
5MSOP	2.8 ± 1.2 c	2.8 ± 1.1 c	8.6 ± 3.4 a	4.6 ± 1.9 b	2.4 ± 0.7 c	1.2 ± 0.5 d	1.7 ± 1.2 d	3.3 ± 2.9 c	4.3 ± 1.7 b	5.5 ± 2.4 b	4.0 ± 1.3 b	3.7 ± 0.9 b
5MTP	0.5 ± 0.5	0.2 ± 0.1	–	–	–	–	–	–	–	–	–	–
Benzyl	*0.1 ± 0.1*	2.0 ± 1.1 a	2.6 ± 0.7 a	2.4 ± 0.7 a	0.8 ± 0.2 b	0.6 ± 0.3 bc	0.6 ± 0.3 bc	0.6 ± 0.2 bc	0.5 ± 0.1 c	0.6 ± 0.2 bc	0.5 ± 0.2 bc	0.6 ± 0.1 bc
2PE	4.1 ± 2.3 a	3.0 ± 1.7 ab	*0.4 ± 1.1*	0.7 ± 0.6 c	0.6 ± 0.6 c	1.0 ± 1.5 c	0.1 ± 0.2 c	–	–	0.1 ± 0.2 c	0.8 ± 0.4 c	1.0 ± 0.6 bc
I3M	2.2 ± 0.6 c	5.5 ± 1.9 b	8.4 ± 2.7 a	0.6 ± 0.2 d	0.4 ± 0.2 d	0.2 ± 0.1 f	0.2 ± 0.0 f	0.2 ± 0.0 e	0.2 ± 0.0 e	0.2 ± 0.1 f	0.2 ± 0.1 f	0.2 ± 0.0 e
4OHI3M	1.2 ± 0.3 b	0.9 ± 0.3 b	0.5 ± 0.8 c	*0.0 ± 0.0*	4.4 ± 1.7 a	0.2 ± 0.2 c	*0.0 ± 0.0*	–	–	–	–	–
4MOI3M	1.1 ± 0.5 b	0.9 ± 0.6 bc	3.1 ± 0.9 a	–	0.4 ± 0.3 c	*0.0 ± 0.0*	*0.0 ± 0.0*	–	–	–	–	*0.0 ± 0.0*
1MOI3M	0.8 ± 0.8 b	8.7 ± 6.3 a	*0.0 ± 0.0*	0.5 ± 0.2 b	0.2 ± 0.2 c	0.1 ± 0.2 c	*0.0 ± 0.0*	*0.1 ± 0.1*	*0.0 ± 0.0*	*0.0 ± 0.0*	–	0.1 ± 0.1 c

1 *Given in percent of the total GLS content in each sample. –, not detected*.

2 *GLS proportions labeled with different letters are significantly different (details of the statistical analyses are summarized in Supplementary Table S1)*.

3 *if GLS was only detected in one or two samples, the mean percentage is written in italics and data were not included in the statistical analysis. For GLS abbreviations see legend of Table 1*.

To determine whether *P. chrysocephala* can activate sequestered GLS for their own defense, myrosinase activity assays were performed with crude protein extracts of larvae and adults. However, no myrosinase activity was detected under our assay conditions using allyl- and 2-hydroxy-3-butenyl GLS as substrates.

### *P. chrysocephala* Partially Prevent GLS Hydrolysis

To assess how much of the total ingested GLS *P. chrysocephala* adults accumulate in their body, adults were fed for one day with detached leaves of the *A. thaliana myb28* × *myb29* mutant spiked with 250 nmol of 4MSOB GLS. The GLS recovery from control leaves without beetles was 93%, which demonstrates that only a small fraction of GLS was metabolized in leaves under assay conditions. The amount of ingested GLS was determined by quantifying the remaining amount of 4MSOB GLS in fed leaves. The quantification of 4MSOB GLS in adult beetles and feces samples revealed that adults accumulated 17% of the total ingested GLS in their body, and that only low amounts of intact 4MSOB GLS were present in feces samples (Table 3).

**Table 3:** Metabolic fate of ingested 4MSOB GLS in *P. chrysocephala* adults.

	**Mean percentage ± SD (*****N*** **= 6)**	
	**Beetle**	**Feces**
Intact 4MSOB GLS	17.42 ± 2.89	0.94 ± 0.26
Desulfo-4MSOB GLS	6.44 ± 1.08	1.20 ± 0.34
4MSOB-ITC	0.71 ± 0.47	1.46 ± 0.62
4MSOB-cyanide	Not detected	9.33 ± 2.69
4MSOB-amine	1.72 ± 0.49	3.80 ± 0.45
4MSOB-acetamide	0.06 ± 0.02	0.70 ± 0.19
4MSOB-ITC-GSH conjugate	2.59 ± 1.44	0.52 ± 0.18
4MSOB-ITC-CysGly conjugate	0.01 ± 0.01	0.20 ± 0.07
4MSOB-ITC-Cys conjugate	0.12 ± 0.07	0.44 ± 0.22
4MSOB-ITC-Cyclic-Cys conjugate A	0.02 ± 0.01	1.67 ± 0.85
4MSOB-ITC-Cyclic-Cys conjugate C	0.84 ± 1.01	10.78 ± 9.54
Total^a^	60.96 ± 11.19	

a *The total of 61% corresponds to the sum of all quantified metabolites in bodies and feces of P. chrysocephala relative to the total amount of ingested 4MSOB GLS calculated from the remaining 4MSOB GLS amount per leaf after adult feeding. 4MSOB-Cyclic-Cys conjugate A, 2-(4-(methylsulfinyl)butylamino)-4,5-dihydrothiazole-carboxylic acid; 4MSOB-ITC-Cyclic-Cys conjugate C, 4-hydroxy-3-(4-(methylsulfinyl)butyl)-2-thioxothiazolidine-4-carboxylic acid*.

In addition to the intact GLS, desulfo-4MSOB GLS was present in body and feces samples, and this metabolite accounted for about 8% of the total GLS *P. chrysocephala* ingested (Table 3). Because GLS sequestration and desulfation explained the fate of only 26% of the total ingested GLS, we hypothesized that most ingested GLS are hydrolyzed by the plant myrosinase during feeding.

**Figure 3:**
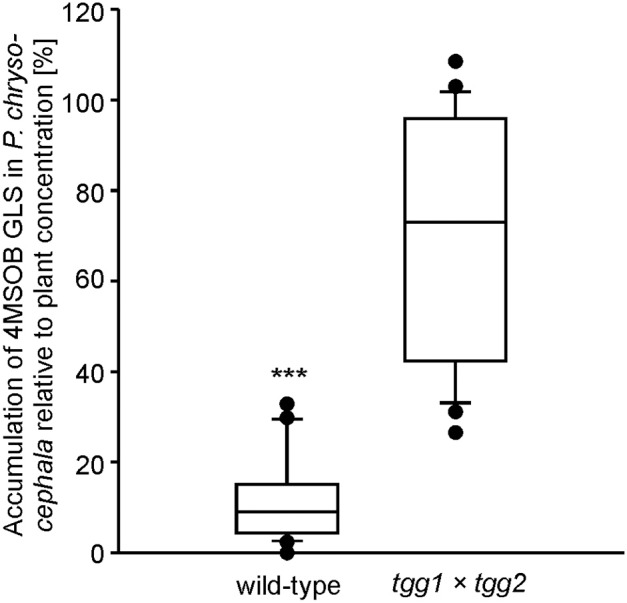
Amount of intact 4-methylsulfinylbutyl (4MSOB) GLS in *P. chrysocephala* adults after feeding on the myrosinase-deficient mutant (*tgg1* × *tgg2*) or wild-type *A. thaliana* Col-0. Two newly emerged adults were allowed to feed on a detached leaf from a five-week old mutant or wild-type plant for 24 h, and subsequently on a detached leaf of the *A. thaliana myb28* × *myb29* mutant devoid of aliphatic GLS for 20 h. Extracted GLS from leaves and adults were analyzed by HPLC-UV. The 4MSOB GLS concentration per mg fresh weight in adults was determined and expressed relative to the 4MSOB GLS concentration in the corresponding leaf which was set to 100% (*N* = 24). ^***^P < 0.001.

To further test the potential influence of plant myrosinase activity on the metabolic fate of GLS in *P. chrysocephala*, we compared the accumulation of 4MSOB GLS in adults that fed on leaves of the myrosinase-deficient *A. thaliana tgg1* × *tgg2* DKO mutant and the corresponding wild-type, respectively. Although adults fed equally on both *A. thaliana* genotypes (*N* = 27, Student's *t*-test, *t* = −0.53, *P* = 0.60) containing similar concentrations of 4MSOB GLS (*N* = 24, Student's *t*-test, *t* = 0.45, *P* = 0.66), beetles contained six times more 4MSOB GLS after feeding on *tgg1* × *tgg2* leaves than after feeding on leaves of wild-type plants (*N* = 24; Mann-Whitney *U*-test, *U* = 4.0, *P* < 0.001; Figure 3).

### *P. chrysocephala* Detoxify 4MSOB-ITC by Conjugation to Glutathione

**Figure 4:**
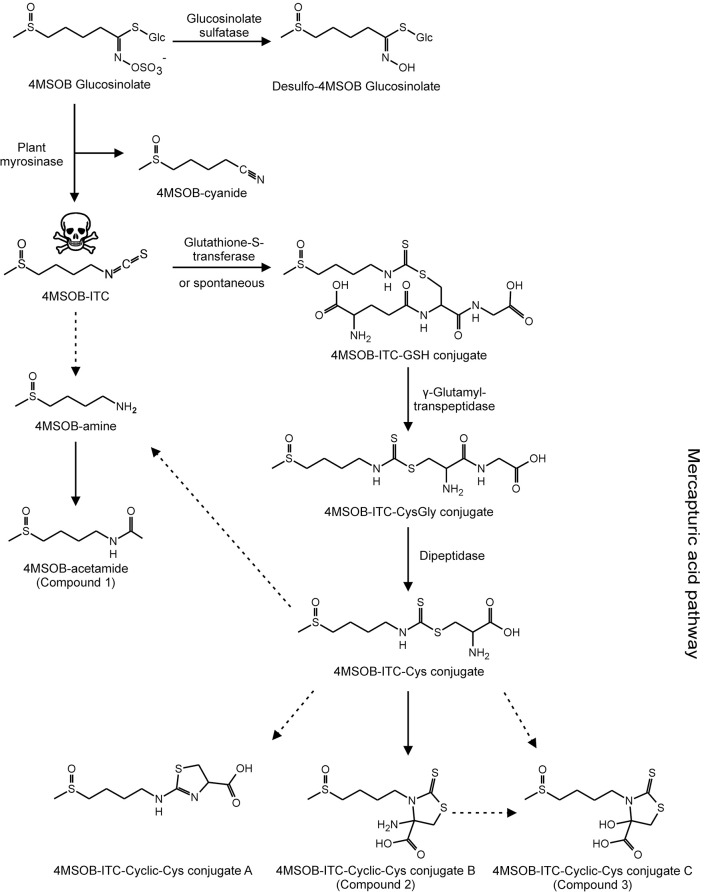
Metabolic fate of ingested 4MSOB GLS in *P. chrysocephala* adults. Ingested 4MSOB GLS is partially sequestered and additionally detoxified by desulfation. A fraction of ingested 4MSOB GLS is activated by the plant myrosinase to 4MSOB isothiocyanate (ITC) and the corresponding nitrile (4MSOB-cyanide). 4MSOB-ITC is detoxified by conjugation with glutathione (GSH). The ITC-GSH conjugate is metabolized via the mercapturic acid pathway to several cyclic ITC-Cys conjugates, i.e., 2-(4-(methylsulfinyl)butylamino)-4,5-dihydrothiazole-carboxylic acid (4MSOB-ITC-Cyclic-Cys conjugate A), 4-amino-3- (4-(methylsulfinyl)butyl)-2- thioxothiazolidine-4-carboxylic acid (4MSOB-ITC-Cyclic-Cys conjugate B, compound 2), and 4-hydroxy-3-(4-(methylsulfinyl)butyl)-2-thioxothiazolidine-4-carboxylic acid (4MSOB-ITC-Cyclic-Cys conjugate C, compound 3). Additionally, 4MSOB-amine and 4MSOB-acetamide (compound 1) were detected. Proposed metabolic steps are indicated with dashed arrows.

In *A. thaliana* Col-0 wild-type leaves, tissue damage leads to the conversion of 4MSOB GLS to 4MSOB-ITC and minor amounts of the corresponding nitrile (Lambrix et al., 2001; Schramm et al., 2012). These expected GLS breakdown products were also present in our samples, and accounted for 2% and 9% of the total ingested GLS, respectively (Table 3). In agreement with previous studies on the metabolism of dietary ITCs in insect and molluscan herbivores (Schramm et al., 2012; Falk et al., 2014; Gloss et al., 2014; Jeschke et al., 2017), we found several 4MSOB-ITC-derived metabolites which resulted from the conjugation with GSH, i.e., ITC conjugates with GSH, CysGly and Cys, respectively. In addition, the cyclic Cys conjugate 2-(4-(methylsulfinyl)butylamino)-4,5-dihydrothiazole-carboxylic acid was detected, a metabolite that was previously discovered in the feces of molluscs (Falk et al., 2014) (Figure 4). The NAC conjugate of 4MSOB-ITC was not detected in *P. chrysocephala*. Together, these metabolites accounted for the metabolic fate of 6% of the total ingested GLS (Table 3). Moreover, ITC-GSH conjugates can be metabolized to amines and raphanusamic acid (Bednarek et al., 2009; Piślewska-Bednarek et al., 2018). Indeed, 4MSOB-amine was present in *P. chrysocephala* body and feces extracts and accounted for 5.5% of the total ingested GLS (Table 3; Supplementary Figure S1).

### Identification of Previously Unknown Metabolites Derived From 4MSOB-ITC in Feces of *P. chrysocephala*

To search for other 4MSOB-ITC-derived metabolites, feces extracts of beetles that had fed on detached leaves of different *A. thaliana* genotypes (Col-0 wild-type, *myb28* × *myb29* lacking aliphatic GLS, 4MSOB GLS-spiked *myb28* × *myb29*, and myrosinase-deficient *tgg1* × *tgg2*) were compared by LC-MS. Peaks that were only detected when adults had fed on *A. thaliana* wild-type and 4MSOB GLS-spiked *myb28* × *myb29* leaves were further analyzed with high resolution mass spectrometry (HRMS) and MS/MS. This approach led to the identification of three previously unknown 4MSOB-ITC-derived metabolites, which were isolated from feces extracts by preparative HPLC to elucidate their structure with NMR.

Compound 1 showed a molecular ion peak of *m/z* 178.09012 ([M+H]^+^) which was consistent with the molecular formula C_7_H_15_O_2_NS ((C_7_H_15_O_2_NS+H)^+^ calculated (calcd) *m/z* 178.089625). NMR analyses revealed that this compound is 4MSOB-acetamide (Supplementary Table S3), and excreted and chemically synthesized 4MSOB-acetamide had the same LC retention time and mass spectrum (Supplementary Figure S2). However, the quantification of 4MSOB-acetamide by LC-MS/MS revealed that it was only a minor metabolite, accounting for >1% of the total ingested GLS in *P. chrysocephala* (Table 3).

**Figure 5:**
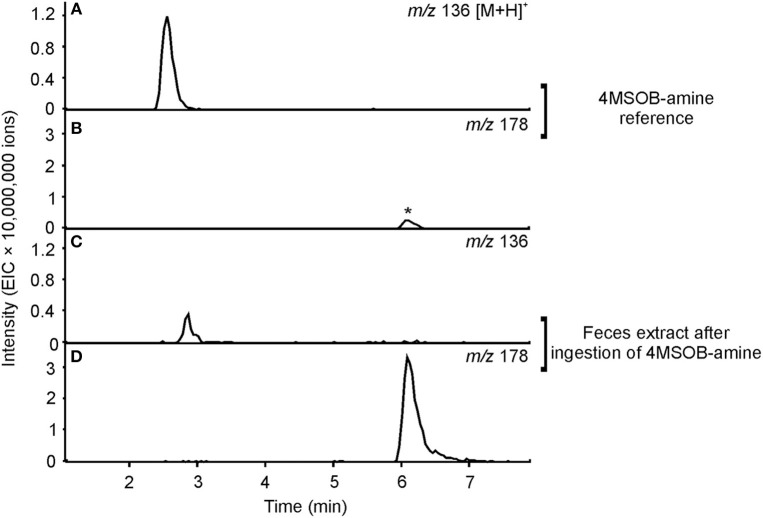
Metabolism of 4MSOB-amine in *P. chrysocephala*. Feces of adults which ingested 4MSOB-amine and subsequently fed on a detached leaf of the *A. thaliana myb28* × *myb29* mutant devoid of aliphatic GLS for 20 h were extracted with 0.1% (v/v) formic acid (pH 3). Feces extracts were analyzed by HPLC-MS (ion trap) in comparison to the 4MSOB-amine reference. Extracted ion chromatograms of 4MSOB-amine (*m/z* 136; **A,C**) and 4MSOB-acetamide (*m/z* 178; **B**,**D**) in positive ionization mode [M+H]^+^ are shown. A low amount of 4MSOB-acetamide was detected in the 4MSOB-amine reference (peak labeled with an asterisk).

To elucidate whether *P. chrysocephala* converts 4MSOB-amine to 4MSOB-acetamide *in vivo*, adults were fed aqueous 4MSOB-amine or water as a control. Afterwards, beetles were allowed to feed on *A. thaliana myb28* × *myb29* leaves to obtain feces for chemical analysis. After ingestion of 4MSOB-amine, adults excreted 4MSOB-acetamide and only traces of 4MSOB-amine, whereas both metabolites were not present in feces of adults, which had ingested water as a control (Figure 5).

**Figure 6:**
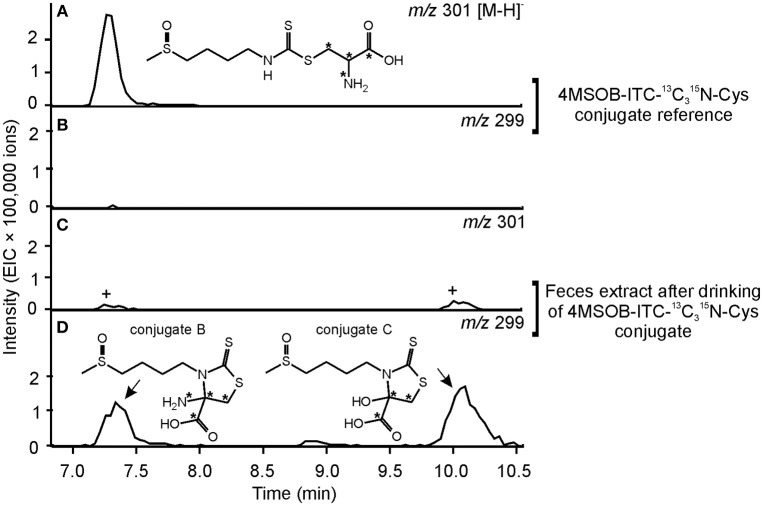
Metabolism of 4MSOB-ITC-^13^${\text{C}}_{3}^{15}$N-Cys in *P. chrysocephala*. Feces of adults which ingested 4MSOB-ITC-^13^${\text{C}}_{3}^{15}$N-Cys conjugate and subsequently fed on a detached leaf of the *A. thaliana myb28* × *myb29* mutant devoid of aliphatic GLS for 20 h were extracted with 0.1% (v/v) formic acid (pH 3). Feces extracts were analyzed by HPLC-MS (ion trap) in comparison to the 4MSOB-ITC-^13^C_3_^15^N-Cys conjugate reference. Extracted ion chromatograms of 4MSOB-ITC-^13^C^15^N-Cys conjugate (*m/z* 301; **A,C**) and the labeled 4-amino-3-(4-(methylsulfinyl)butyl)-2-thioxothiazolidine-4-carboxylic acid (4MSOB-ITC-Cyclic-Cys conjugate B), and 4-hydroxy-3-(4-(methylsulfinyl)butyl)-2-thioxothiazolidine-4-carboxylic acid (4MSOB-ITC-Cyclic-Cys conjugate C) (*m/z* 299; **B,D**) in negative ionization mode [M–H]^−^ are shown. 4MSOB-ITC-^13^${\text{C}}_{3}^{15}$N-Cys conjugate and 4MSOB-ITC-Cyclic-Cys conjugate B have the same retention time under the applied HPLC conditions. The peaks marked with “+” correspond to the ^34^S-isotopologues of the labeled 4MSOB-ITC-Cyclic-Cys conjugates B and C.

Compound 2 showed a molecular ion peak of *m/z* 295.02459 ([M-H]^−^) which was consistent with the molecular formula C_9_H_16_O_3_N_2_S_3_ ((C_9_H_16_O_3_N_2_S_3_-H)^−^ calcd *m/z* 295.025026). Tandem MS and NMR analyses revealed that compound 2 is a conjugate of 4MSOB-ITC which was assigned as 4-amino-3-(4-(methylsulfinyl)butyl)-2-thioxothiazolidine-4-carboxylic acid (4MSOB-ITC-Cyclic-Cys conjugate B; Supplementary Figure S3; Supplementary Table S4). During isolation by SPE and preparative HPLC, compound 2 was unstable and partially converted to compound 3, which indicated that both metabolites are related. Compound 3 showed a molecular ion peak of *m/z* 296.00901 ([M-H]^−^) which was consistent with the molecular formula C_9_H_15_O_4_NS_3_ ((C_9_H_15_O_4_NS_3_-H)^−^ calcd *m/z* 296.00904). The structure of compound 3 was very similar to compound 2 and was assigned as 4-hydroxy-3-(4-(methylsulfinyl)butyl)-2-thioxothiazolidine-4-carboxylic acid (4MSOB-ITC-Cyclic-Cys conjugate C) according to LC-MS/MS and NMR analyses and by comparison to a chemically synthesized standard (Supplementary Figure S4; Supplementary Table S5). Mercaptopyruvic acid conjugates of benzenic ITCs were previously described in guinea-pigs, rabbits, and mice, and were shown to be derived from the ITC-Cys conjugate (Görler et al., 1982; Eklind et al., 1990). To test whether compounds 2 and 3 are derived from the ITC-Cys conjugate in *P. chrysocephala*, we performed a feeding experiment with the stable isotope labeled ^13^${\text{C}}_{3}^{15}$N-Cys conjugate of 4MSOB-ITC (*m/z* 301 [M-H]^−^). The labeled products were expected to have the same mass of the molecular ion (*m/z* 299 [M-H]^−^) because the ^15^N-labeled amino group at the C4-position in compound 2 is replaced with a hydroxyl group in compound 3 resulting in the loss of one labeled atom. Both 4MSOB-ITC conjugates were detected at the expected retention times of 7.1 min (compound 2) and 10.3 min (compound 3) with *m/z* 299 ([M-H]^−^) in feces extracts by LC-MS, confirming that they were derived from the labeled 4MSOB-ITC-Cys conjugate (Figure 6). These compounds were not detected in feces samples of beetles that ingested water as a control. Compounds 2 and 3 were thus named 4MSOB-ITC-Cyclic-Cys conjugate B and C, respectively (Figure 4).

4MSOB-ITC-Cyclic-Cys conjugate B was not quantified because there was no synthetic standard available. The relative proportion of 4MSOB-ITC-Cyclic-Cys conjugate C in samples from the quantitative feeding experiment varied considerably and accounted for 11.6 ± 9.0% of the total ingested GLS (*N* = 6; Table 3).

All quantified derivatives of the mercapturic acid pathway together, including these novel metabolites, accounted for the metabolic fate of 17% of the total ingested GLS in our feeding experiment.

## Discussion

The cabbage stem flea beetle, *P. chrysocephala*, is adapted to crucifer plants that are defended with the GLS-myrosinase system. Because feeding damage usually causes rapid enzymatic breakdown of GLS to toxic ITCs in the insect gut, we tested the hypothesis that *P. chrysocephala* can prevent GLS hydrolysis during feeding. Previously, we discovered a strong accumulation of GLS in the closely related flea beetle *P. striolata*, showing that adults at least partially prevent GLS activation in ingested plant tissue (Beran et al., 2014). The presence of GLS in all life stages of *P. chrysocephala* indicated a similar mechanism in this flea beetle species, but according to our quantitative analysis, less than 20% of the ingested 4MSOB GLS remained intact in adults. In addition, a minor proportion of 4MSOB GLS is metabolized by desulfation, a strategy that prevents enzymatic activation of GLS and requires activity of a sulfatase enzyme toward GLS. Such GLS sulfatase activity is known to be present in several specialist as well as generalist herbivores (Ratzka et al., 2002; Falk and Gershenzon, 2007; Opitz et al., 2011; Malka et al., 2016), but was not detected in *P. striolata* (Beran et al., 2014). Our results thus suggest that *P. chrysocephala* also possess GLS sulfatase activity. Compared to other adapted herbivores such as *A. rosae* and *P. xylostella* (Ratzka et al., 2002; Müller and Wittstock, 2005; Opitz et al., 2011; Jeschke et al., 2017), the proportion of sequestered and desulfated GLS in *P. chrysocephala* is low and thus does not prevent exposure to GLS hydrolysis products during feeding.

The ability to prevent GLS hydrolysis is not a prerequisite to feed or specialize on crucifers because herbivores may use other mechanisms to avoid ITC formation or toxicity. For example, cabbage white butterfly larvae excreted more than 90% of ingested 4MSOB GLS as the less toxic nitrile due to their gut-expressed NSP, whereas in other insects which do not possess endogenous NSP activity, 4MSOB GLS was mainly hydrolyzed to ITCs with only 9–15% nitrile (Gloss et al., 2014; Jeschke et al., 2017). In our quantitative study, the nitrile accounted for 9%, which also suggests the activation of a major proportion of ingested 4MSOB GLS (~65%) to ITCs. On the other hand, herbivory can induce the expression of NSP in rosette leaves of *A. thaliana* Col-0 leading to a higher percentage of the simple nitrile upon 4MSOB GLS hydrolysis (Burow et al., 2009). Indeed, free ITCs were present in low amounts (~2%), whereas conjugates of ITCs accounted for at least 17% of the metabolic fate of ingested GLS. This result confirms ITC formation and shows that *P. chrysocephala* metabolizes ITCs via the mercapturic acid pathway after conjugation to GSH. The conserved mercapturic pathway plays a key role in ITC metabolism for example in generalist lepidopteran larvae, but this detoxification strategy is associated with a high metabolic cost due to the loss of amino acids, in particular Cys (Schramm et al., 2012; Gloss et al., 2014; Jeschke et al., 2016, 2017).

In addition to the known GSH, CysGly, and Cys conjugates of 4MSOB-ITC, three different cyclic derivatives of Cys-conjugate were identified in *P. chrysocephala*. One of these (conjugate A) was previously detected in molluscs (Falk et al., 2014), whereas 4-amino-3-(4-(methylsulfinyl)butyl)-2-thioxothiazolidine-4-carboxylic acid (conjugate B) and 4-hydroxy-3-(4-(methylsulfinyl)butyl)-2-thioxothiazolidine-4-carboxylic acid (conjugate C; Figures 4, 6) are described for the first time. The formation of cyclic mercaptopyruvic acid conjugates (conjugate C) with other ITCs was previously reported in rodents (Görler et al., 1982; Eklind et al., 1990). The presence of the cyclic conjugates B and C in *P. chrysocephala* hints at a specific metabolism of ITCs in this beetle, because these conjugates were not detected in feeding studies with lepidopteran and dipteran herbivores using radiolabeled 4MSOB GLS (Gloss et al., 2014; Jeschke et al., 2017). Furthermore, the NAC conjugate of 4MSOB-ITC, a major product of ITC metabolism in *S. flava* (Gloss et al., 2014), was only found in traces in *P. chrysocephala*.

While the conversion of the ITC-Cys conjugate to the cyclic conjugate A can occur non-enzymatically (Kawakishi and Namiki, 1982), the formation of conjugates B and C is most likely enzymatic. Görler et al. proposed that the ITC-Cys conjugate is transaminated to the *S*-substituted mercaptopyruvate which cyclizes spontaneously following enolization to the thiazolidinethione (conjugate C). In *P. chrysocephala*, conjugate B may be formed by oxidation of the amino-group, and convert spontaneously to conjugate C as observed during the purification of conjugate B from feces extracts. It also remains an open question whether the cyclization enhances stability of the ITC conjugate compared to the thiocarbamoyl adduct which is known to be unstable under physiological conditions (Bruggeman et al., 1986; Nakamura et al., 2009).

We identified two other minor metabolites in *P. chrysocephala* that have so far not been reported as products of GLS metabolism in insects, i.e., 4MSOB-amine and its acetylated derivative 4MSOB-acetamide. By feeding adults with 4MSOB-amine, we demonstrated the conversion of 4MSOB-amine to 4MSOB-acetamide *in vivo* (Figure 5), but the precursor of 4MSOB-amine in *P. chrysocephala* is so far unknown. For example, amines may arise by non-enzymatic degradation of ITCs in aqueous solutions (Pechacek et al., 1997; Negrusz et al., 1998). In our experiments, 4MSOB-ITC remained stable in the extraction solvent *in vitro*, but might degrade to the amine in the beetle gut. In *A. thaliana* plants, the formation of indol-3-ylmethylamine depends on the conjugation of indol-3-ylmethyl-ITC to GSH and thus is a product of the mercapturic acid pathway (Bednarek et al., 2009; Piślewska-Bednarek et al., 2018). Furthermore, specific hydrolase enzymes may catalyze hydrolysis of ITCs to amines. Such an ITC hydrolase is for example present in a *Pectobacterium* strain that was isolated from the larval gut of the cabbage root fly, *Delia radicum*, where it possibly contributes to the detoxification of ITCs (Welte et al., 2016a,b). We currently investigate the possible role of the gut microbiome in ITC detoxification and formation of amines in *P. chrysocephala*.

Based on our results, it is unclear whether the conjugation to GSH represents the major detoxification strategy for dietary ITCs, because the metabolic fate of about 40% of the total ingested GLS remains unknown. Therefore, we cannot exclude the possibility that *P. chrysocephala* uses additional strategies to metabolize ITCs, nitriles, GLS, or desulfo-GLS. Feeding studies with radiolabeled GLS enable the identification of novel metabolites and are generally better suited to analyze the metabolic fate of ingested GLS because each compound can be quantified directly (Opitz et al., 2011; Gloss et al., 2014; Jeschke et al., 2017). Unfortunately, this approach failed with *P. chrysocephala*, because the amount of ingested radiolabeled 4MSOB GLS was too low. To clarify the importance of the mercapturic acid pathway in the detoxification of ITCs, we plan to manipulate this pathway via RNAi to analyze effects on ITC detoxification and fitness. Potential targets include glutathione *S*-transferases that catalyze the first step of the mercapturic acid pathway, although the conjugation of ITCs to GSH can also proceed non-enzymatically.

Independent of the fact that a relatively small proportion of ingested 4MSOB GLS was sequestered, the presence of GLS in all life stages of *P. chrysocephala* shows that this specialist is adapted to GLS. Both *P. chrysocephala* larvae and adults selectively sequester 2-hydroxy-3-butenyl GLS from their host plant *B. rapa*, but interestingly, the GLS composition of eggs differed from that of all other life stages and was more similar to that of *B. rapa*. For example, the proportion of indol-3-ylmethyl GLS in eggs was 42 times higher than in adults, suggesting an active transport mechanism for GLS into eggs. The presence of indolic GLS in eggs also demonstrates the uptake of indolic GLS from the gut into the body of adults, but nevertheless, GLS uptake may be selective and dependent on the GLS structure in *P. chrysocephala*. For example, the preferred uptake of 2-hydroxy-3-butenyl GLS compared to 3-butenyl GLS may lead to the observed GLS sequestration pattern in larvae and adults (Figure 2; Table 2). On the other hand, similar uptake rates for both GLS followed by enzymatic hydroxylation of 3-butenyl GLS would also change the relative proportions of both GLS. However, considering that 2-hydroxy-3-butenyl- and 3-butenyl GLS together account for less than 50% of the total GLS in *B. rapa*, but for up to 76% in *P. chrysocephala* adults (Table 2), enzymatic hydroxylation of 3-butenyl GLS alone would not explain the high proportion of 2-hydroxy-3-butenyl GLS in adults.

Interestingly, the flea beetle *P. striolata* selectively accumulates aliphatic GLS to ten-fold higher concentrations than *P. chrysocephala* (Beran et al., 2014), which indicates that the mechanism or efficiency of GLS uptake or turnover differs between both species. Moreover, *P. striolata* may use sequestered GLS for its defense against natural enemies as they express their own myrosinase (Beran et al., 2014), whereas enzyme assays with *P. chrysocephala* did not reveal endogenous myrosinase activity. Thus, the function of sequestered GLS in *P. chrysocephala* remains ambiguous.

Notably, the two flea beetle genera *Phyllotreta* and *Psylliodes* that use GLS-containing plants as hosts differ remarkably in their host plant range. While more or less all *Phyllotreta* species are specialized on crucifers, *Psylliodes* species are associated with host plants belonging to 27 different plant families (Jolivet and Hawkeswood, 1995). For example, about 65% of the middle European *Psylliodes* species feed on GLS-containing plants, while other species are specialized to feed on grasses or solanaceous plants that contain distinct defense compounds (Mohr, 1966). Thus, to answer the question how this complex GLS metabolism evolved in *P. chrysocephala*, a robust phylogeny of this genus and the subfamily Galerucinae is needed. Although some progress has been made on the phylogeny of the Galerucinae, the relationships at genus level are not well-resolved; yet, based on the available data it seems unlikely that *Phyllotreta* and *Psylliodes* are sister genera (Ge et al., 2012; Nie et al., 2018). Furthermore, the observed differences regarding GLS sequestration, insect myrosinase and GLS desulfation support the hypothesis that *Phyllotreta* and *Psylliodes* adapted independently from each other to GLS.

## Conclusion

Most crucifer-specialist herbivores studied so far developed efficient strategies to prevent the breakdown of dietary GLS to reactive defense metabolites. Although the cabbage stem flea beetle selectively sequesters aliphatic GLS and additionally converts GLS to stable desulfo-GLS, this specialist does not avoid GLS hydrolysis completely. Instead, *P. chrysocephala* detoxifies ITCs by conjugation with glutathione via the conserved mercapturic acid pathway. From an evolutionary perspective, the utilization of a common detoxification pathway combined with specialized strategies preventing GLS hydrolysis is intriguing. Based on our results, we hypothesize that the ability to deactivate ITCs by conjugation with glutathione was a prerequisite to survive on crucifer plants, and subsequently enabled the evolution of GLS sequestration and detoxification mechanisms preventing ITC formation in crucifer-feeding *Psylliodes* species. At this background, we propose the genus *Psylliodes* as an excellent model to investigate molecular adaptations to chemical plant defenses that facilitate host shifts and thus drive the species diversification of herbivorous insects.

## Author Contributions

FBer, CP, S-JA, SB, and MR conceived and designed the experiments. FBer, TS, CP, S-JA, FBet, AS, DV, SL, and MR performed the experiments. FBer, TS, CP, S-JA, DV, SB, SL, and MR analyzed the data. FBer and GK performed the statistical analysis. FBer wrote the manuscript. All authors commented on the manuscript.

### Conflict of Interest Statement

The authors declare that the research was conducted in the absence of any commercial or financial relationships that could be construed as a potential conflict of interest.
