# Supplementary material for: One Pathway Is Not Enough: The Cabbage Stem Flea Beetle Psylliodes chrysocephala Uses Multiple Strategies to Overcome the Glucosinolate-Myrosinase Defense in Its Host Plants
Source: Front Plant Sci. 2018 Dec 7;9:1754. doi: 10.3389/fpls.2018.01754 (PMC6292997; doi:10.3389/fpls.2018.01754)
Supplement: Supplementary file 1 [file Table_1.docx]

**Supplementary Table S1.** Methods and results of statistical analysis of total glucosinolate (GLS) concentration and GLS amount per individual in all life stages of *P. chrysocephala*, and GLS proportions in *B. rapa* leaves and *P. chrysocephala* life stages.

| **GLS** | **Transformation** | **Method** | **Statistics** | ***P*-value** |
| --- | --- | --- | --- | --- |
| Total concentration | - | generalized least squares | *LR* = 51.02 | < 0.001 |
| Amount per individual | - | generalized least squares | *LR* = 146.45 | < 0.001 |
| 3But | arcsin-square-root | generalized least squares | *LR* = 70.68 | < 0.001 |
| 4Pent | arcsin-square-root | generalized least squares | *LR* = 99.47 | < 0.001 |
| 2OH3But | arcsin-square-root | ANOVA | *F* = 67.73 | < 0.001 |
| 2OH4Pent | - | generalized least squares | *LR* = 98.32 | < 0.001 |
| 5MSOP | arcsin-square-root | generalized least squares | *LR* = 61.90 | < 0.001 |
| 5MTP | - | generalized least squares | *LR* = 2.55 | = 0.110 |
| Benzyl | - | gamma generalized linear model | *D* = -32.46 | < 0.001 |
| 2PE | arcsin-square-root | ANOVA | *F* = 12.08 | < 0.001 |
| I3M | arcsin-square-root | generalized least squares | *LR* = 114.7 | < 0.001 |
| 4OHI3M | arcsin-square-root | generalized least squares | *LR* = 72.06 | < 0.001 |
| 4MOI3M | arcsin-square-root | ANOVA | *LR* = 28.84 | < 0.001 |
| 1MOI3M | arcsin-square-root | generalized least squares | *LR* = 22.03 | < 0.001 |

arcsin, arcus-sinus; LR, likelihood ratio; D, deviance. For abbreviations of GLS see legend of **Table 1**.
