# Supplementary material for: One Pathway Is Not Enough: The Cabbage Stem Flea Beetle Psylliodes chrysocephala Uses Multiple Strategies to Overcome the Glucosinolate-Myrosinase Defense in Its Host Plants
Source: Front Plant Sci. 2018 Dec 7;9:1754. doi: 10.3389/fpls.2018.01754 (PMC6292997; doi:10.3389/fpls.2018.01754)
Supplement: Supplementary file 2 [file Table_2.docx]

**Supplementary Table S2.** LC-MS/MS parameters for multiple reaction monitoring (MRM) of 4-methylsulfinylbutyl (4MSOB) GLS and its derivatives identified in this study on an API3200 mass spectrometer.

| **Compound** | **Q1 [*m/z*]** | **Q3 [*m/z*]** | **DP**  **(V)** | **EP**  **(V)** | **CE**  **(V)** | **CXP**  **(V)** |
| --- | --- | --- | --- | --- | --- | --- |
| intact 4MSOB GLS | 435.9 | 95.8 | -65 | -5 | -60 | 0 |
| desulfo-4MSOB GLS | 358.0 | 196.0 | 30 | 5 | 15 | 5 |
| 4MSOB-ITC | 178.11 | 114 | 26 | 5 | 13 | 4 |
| 4MSOB-cyanide | 146.0 | 129.0 | 38 | 10 | 13 | 4 |
| 4MSOB-amine | 136.0 | 72.0 | 26 | 3 | 17 | 4 |
| 4MSOB-acetamide | 178.0 | 114.0 | 26 | 5 | 15 | 4 |
| 4MSOB-ITC-GSH conjugate | 485.11 | 179.1 | 51 | 5.5 | 29 | 6 |
| 4MSOB-ITC-CysGly conjugate | 356.07 | 136.1 | 21 | 11 | 15 | 4 |
| 4MSOB-ITC-Cys conjugate | 299.06 | 136.1 | 26 | 3 | 15 | 4 |
| 4MSOB-ITC-NAC conjugate | 341.07 | 178.1 | 26 | 3 | 17 | 6 |
| 4MSOB-ITC-Cyclic-Cys conjugate A | 265.11 | 201.0 | 26 | 8.5 | 25 | 4 |
| 4MSOB-ITC-Cyclic-Cys conjugate B | 297.0 | 280.0 | 26 | 3 | 14 | 4 |
| 4MSOB-ITC-Cyclic-Cys conjugate C | 298.0 | 216.0 | 26 | 3 | 17 | 4 |

ITC, isothiocyanate; GSH, glutathione; CysGly, cysteinylglycine; Cys, cysteine; NAC, N-acetylcysteine; 4MSOB-Cyclic-Cys conjugate A, 2-(4-(methylsulfinyl)butylamino)-4,5-dihydrothiazole-carboxylic acid; 4MSOB-ITC-Cyclic-Cys conjugate B, 4-amino-3-(4-(methylsulfinyl)butyl)-2-thioxothiazolidine-4-carboxylic acid; 4MSOB-ITC-Cyclic-Cys conjugate C, 4-hydroxy-3-(4-(methylsulfinyl)butyl)-2-thioxothiazolidine-4-carboxylic acid.
