# Supplementary material for: One Pathway Is Not Enough: The Cabbage Stem Flea Beetle Psylliodes chrysocephala Uses Multiple Strategies to Overcome the Glucosinolate-Myrosinase Defense in Its Host Plants
Source: Front Plant Sci. 2018 Dec 7;9:1754. doi: 10.3389/fpls.2018.01754 (PMC6292997; doi:10.3389/fpls.2018.01754)
Supplement: Supplementary file 3 [file Table_3.docx]

**Supplementary Table S3.** ^1^H- and ^13^C-NMR data of 4MSOB-acetamide isolated from feces of *P. chrysocephala*.

| **Position** | **δ_H_ (*mult*.)** | **δ_C_** | **Structure** |
| --- | --- | --- | --- |
| 1 | 2.80/2.88 (*m*/*m*) | 52.9 | 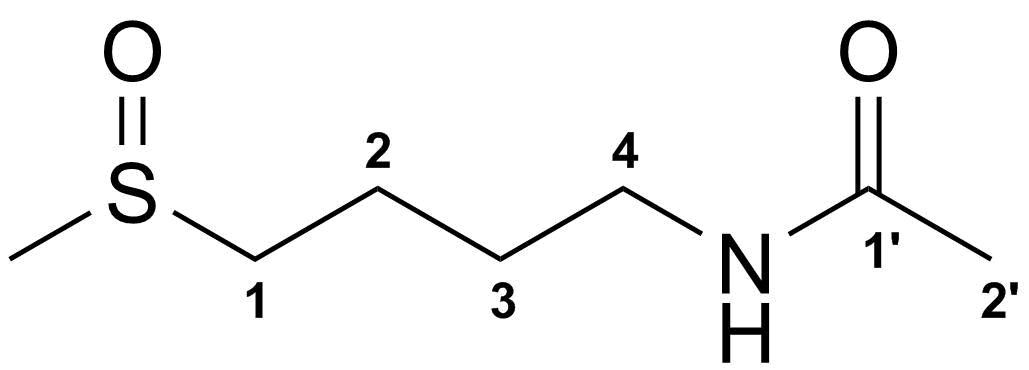 |
| 2 | 1.80 (*m*) | 19.7 |  |
| 3 | 1.68 (*m*) | 28.0 |  |
| 4 | 3.24 (*m*) | 38.4 |  |
| 1' | - | 172.2 |  |
| 2' | 1.96 (*s*) | 21.3 |  |
| -SCH_3_ | 2.66 (*s*) | 36.7 |  |
