# Supplementary material for: One Pathway Is Not Enough: The Cabbage Stem Flea Beetle Psylliodes chrysocephala Uses Multiple Strategies to Overcome the Glucosinolate-Myrosinase Defense in Its Host Plants
Source: Front Plant Sci. 2018 Dec 7;9:1754. doi: 10.3389/fpls.2018.01754 (PMC6292997; doi:10.3389/fpls.2018.01754)
Supplement: Supplementary file 4 [file Table_4.docx]

**Supplementary Table S4.** ^1^H- and ^13^C-NMR data of 4-amino-3-(4-(methylsulfinyl)butyl)-2-thioxothiazolidine-4-carboxylic acid (4MSOB-ITC-Cyclic-Cys conjugate B) isolated from feces of *P. chrysocephala*.

| **Position** | **δ_H_ (*mult*.)** | **δ_C_** | **Structure** |
| --- | --- | --- | --- |
| 1 | 2.82/2.88 (*m*/*m*) | 54.1 | 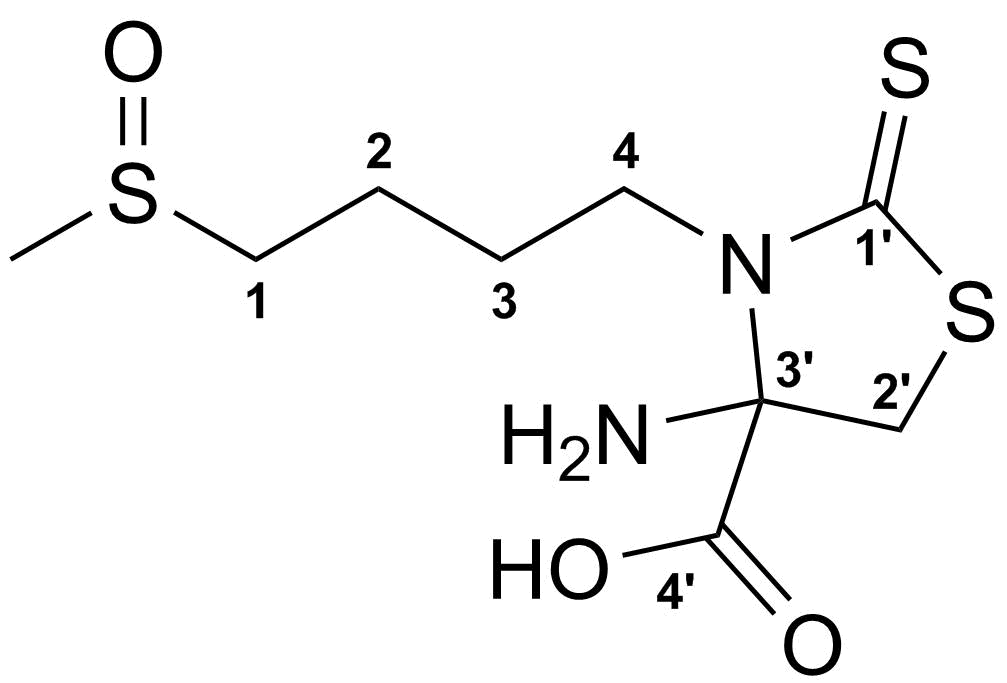 |
| 2 | 1.94 (*m*) | 27.3 |  |
| 3 | 1.76 (*m*) | 21.0 |  |
| 4 | 3.58/3.74 (*m*/*m*) | 46.7 |  |
| 1' | - | 199.0 |  |
| 2' | 3.22/3.99 (*d*, J=11.5 Hz) | 39.7 |  |
| 3' | - | 89.6 |  |
| 4' | - | 174.8 |  |
| -SCH_3_ | 2.63 (*s*) | 37.8 |  |
