# Supplementary figures and images for: One Pathway Is Not Enough: The Cabbage Stem Flea Beetle Psylliodes chrysocephala Uses Multiple Strategies to Overcome the Glucosinolate-Myrosinase Defense in Its Host Plants
Source: Front Plant Sci. 2018 Dec 7;9:1754. doi: 10.3389/fpls.2018.01754 (PMC6292997; doi:10.3389/fpls.2018.01754)

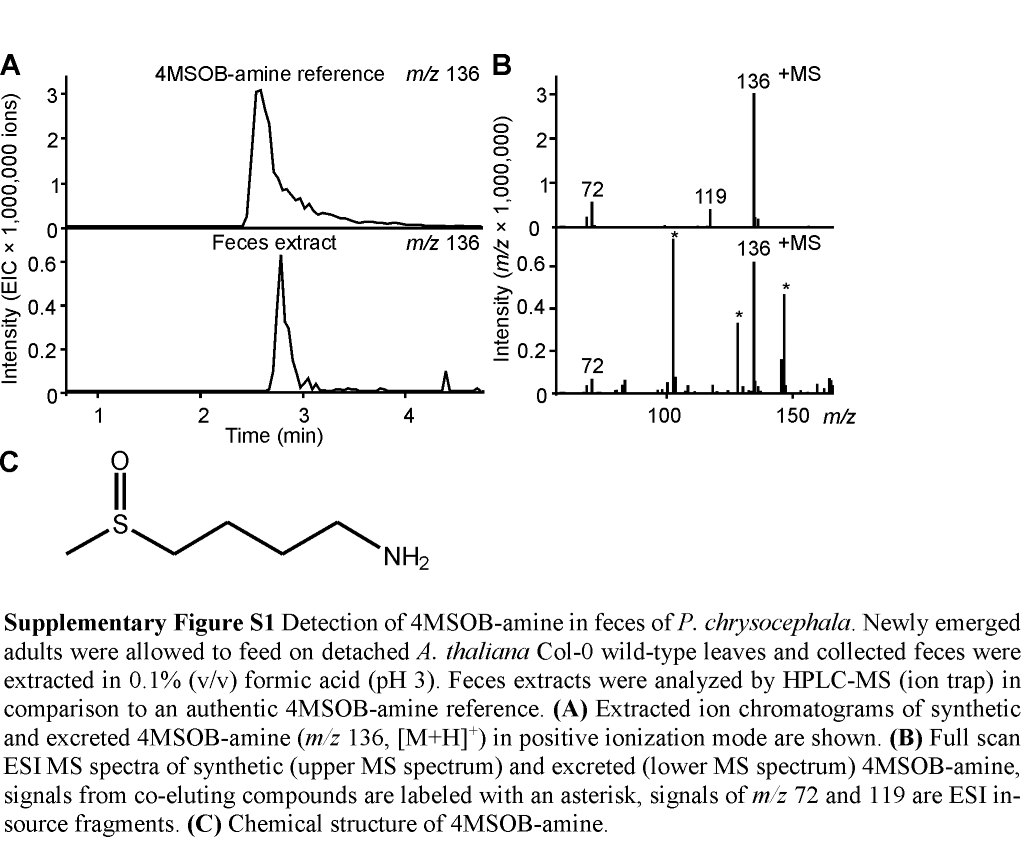

Supplement: Supplementary file 6 [file Image_1.tif]

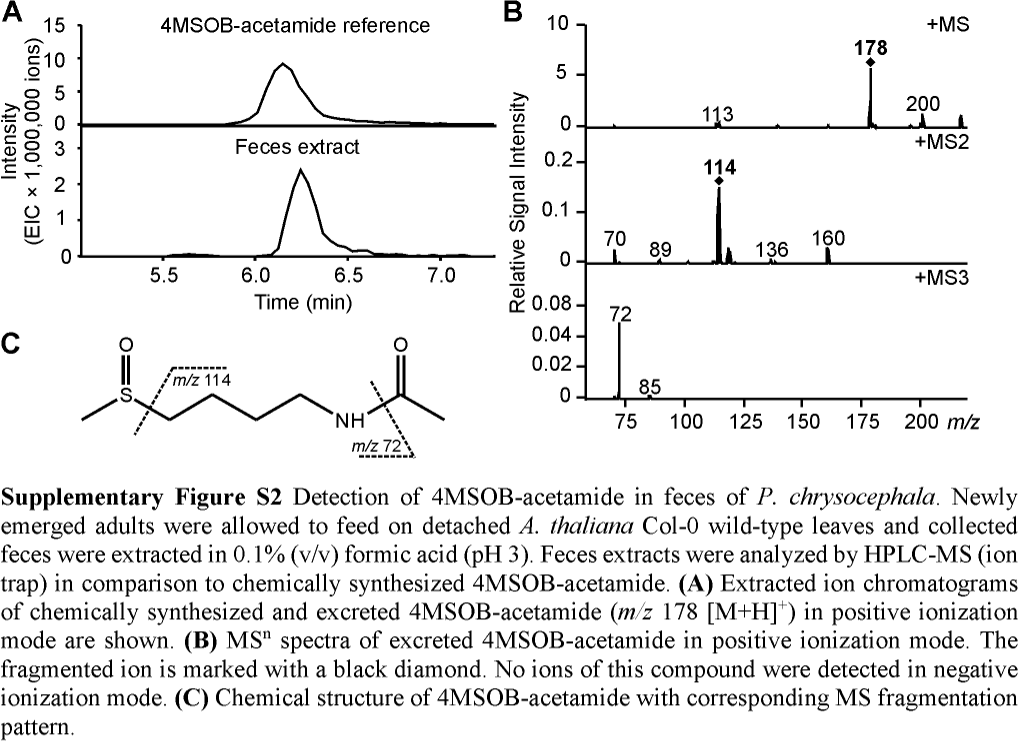

Supplement: Supplementary file 7 [file Image_2.tif]

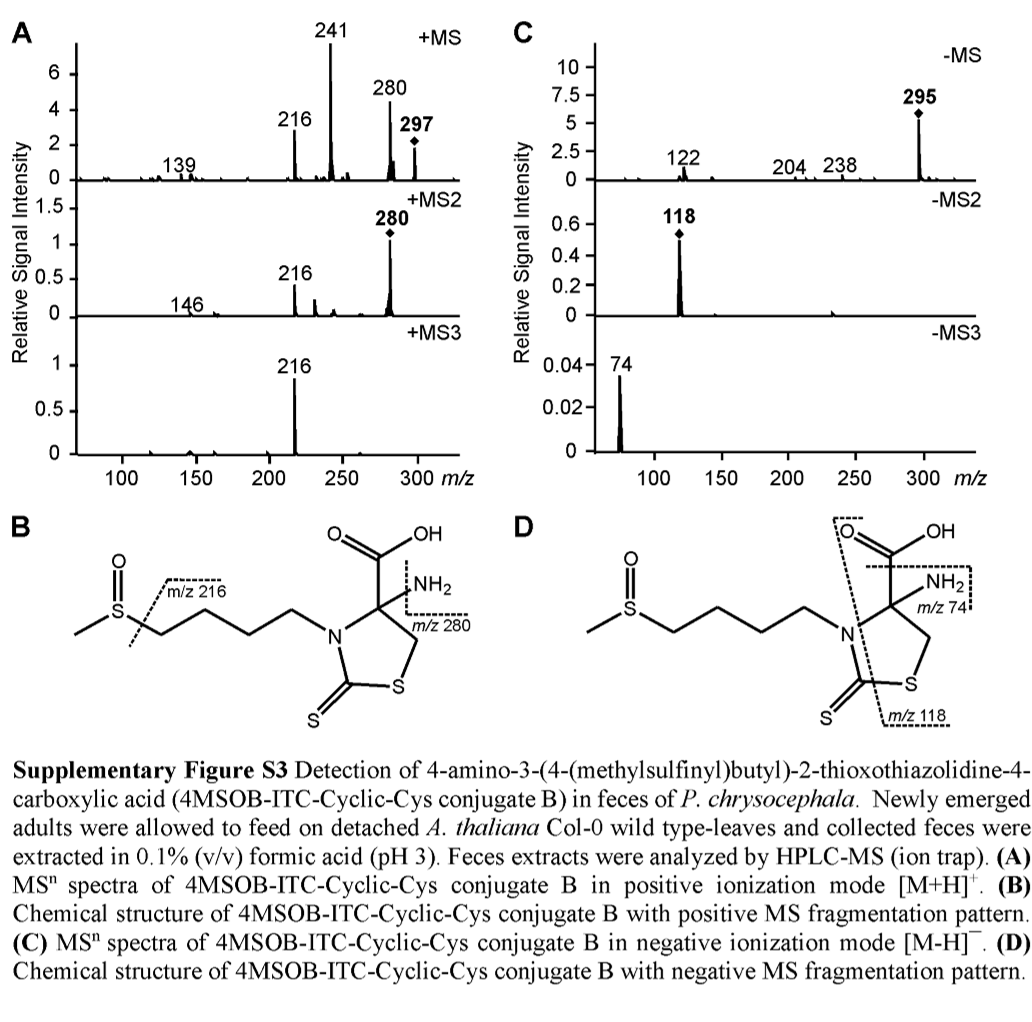

Supplement: Supplementary file 8 [file Image_3.tif]

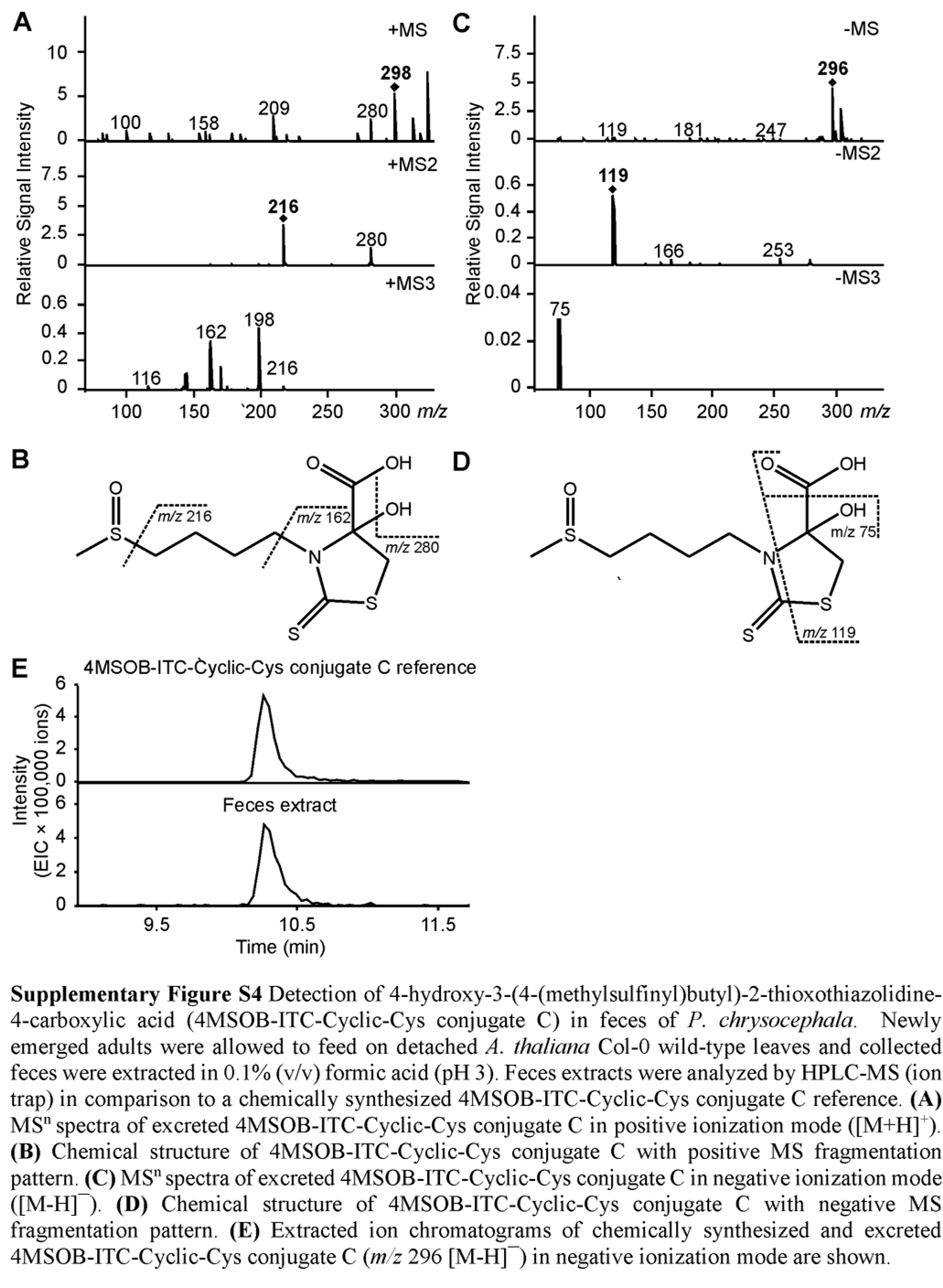

Supplement: Supplementary file 9 [file Image_4.tif]
